# Annual acknowledgement of reviewers

**DOI:** 10.1186/s12862-015-0282-8

**Published:** 2015-02-10

**Authors:** Christopher Foote

**Affiliations:** BioMed Central, Floor 6, 236 Gray′s Inn Road, London, WC1X 8HB UK

## Abstract

The editors of *BMC Evolutionary Biology* would like to thank all our reviewers who have contributed to the journal in Volume 14 (2014).

**Matthew Aardema**

United States of America

**Federico Abascal**

Spain

**Amir Abbasi**

Pakistan

**Patrick Abbot**

United States of America

**Pelayo Acevedo**

Spain

**Cristina Acosta**

Argentina

**Irina Adonina Russian**

Federation

**Kwaku Aduse-Poku**

United Kingdom

**Ingi Agnarsson**

United States of America

**Sebastian Aguilar Pierlé**

United States of America

**Mineaki Aizawa**

Japan

**M.Mar Alba**

Spain

**Fernando Alda**

Spain

**Paulo Amaral**

United Kingdom

**Guruprasad Ananda**

United States of America

**Barbara J Anderson**

New Zealand

**Markus Ankenbrand**

Germany

**Tomohiro Araki**

Japan

**Dan Ardia**

United States of America

**Roberto Argano**

Italy

**Tatiana Arias Garzon**

Hong Kong

**Stephane Aris-Brosou**

Canada

**Sophie Arnaud-Haond**

France

**Birgit Arnholdt-Schmitt**

Portugal

**Natasha Arora**

Switzerland

**Paula Arribas**

Spain

**Nils Arrigo**

Switzerland

**Jairo Arroyave**

United States of America

**Marina Ascunce**

United States of America

**Raquel Assis**

United States of America

**Shreyas Athavale**

United States of America

**Denis Augot**

France

**Stuart Auld**

United Kingdom

**Janelle Ayres**

United States of America

**Mounawer Badri**

Tunisia

**Mumtaz Baig**

India

**Stuart Baird**

France

**Allan Baker**

Canada

**Christopher Balakrishnan**

United States of America

**Daniel Baldassarre**

United States of America

**Janneke Balk**

United Kingdom

**David Baltrus**

United States of America

**Marta Barluenga**

Spain

**Craig Barrett**

United States of America

**Seth Barribeau**

Switzerland

**Aakash Basu**

United States of America

**Andrew Bateman**

Canada

**Fabia Ursula Battistuzzi**

United States of America

**David Baum**

United States of America

**Denis Baurain**

Belgium

**Jeremy Beaulieu**

United States of America

**Lutz Becks**

Germany

**Jasminca Behrmann-Godel**

Germany

**Susanta Behura**

United States of America

**Eric Beitz**

Germany

**Albano Beja-Pereira**

Portugal

**Patrícia Beldade**

Portugal

**Alexa Bely**

United States of America

**John Benzie**

Ireland

**Fernanda Bered**

Brazil

**Stewart Berlocher**

United States of America

**Giorgio Bertorelle**

Italy

**Stephanie Bertrand**

France

**Timothy Bestor**

United States of America

**Kaspar Bienefeld**

Germany

**Jean-Christophe Billeter**

Netherlands

**Olaf Bininda-Emonds**

Germany

**Michael Black**

New Zealand

**John Blazier**

United States of America

**Christoph Bleidorn**

Germany

**Paulette Bloomer**

South Africa

**Leigh Boardman**

South Africa

**Julien Bobe**

France

**Ladislav Bocak**

Czech Republic

**David Bogumil**

Germany

**Kirsten Bohn**

United States of America

**Aureliano Bombarely**

United States of America

**Michael Bonkowski**

Germany

**Camille Bonneaud**

United Kingdom

**Sung Min Boo**

South Korea

**Carlos Andrés Botero**

United States of America

**Jennifer Botha-Brink**

South Africa

**Jean Boubli**

United Kingdom

**Marylene Boulet**

Canada

**Louis Boumans**

Norway

**Laura Boykin**

United States of America

**Robert Bradley**

United States of America

**Hans Breeuwer**

Netherlands

**Amanda Bretman**

United Kingdom

**Jory Brinkerhoff**

United States of America

**Janice Britton-Davidian**

France

**Luciano Brocchieri**

United States of America

**John Brookfield**

United Kingdom

**David Bryant**

New Zealand

**Josef Bryja**

Czech Republic

**Mike Buckley**

United Kingdom

**Martin Burd**

Australia

**John Burka**

Canada

**Geraldine Butler**

Ireland

**Nicholas Butterfield**

United Kingdom

**Nadia Bystriakova**

United Kingdom

**Ana Caicedo**

United States of America

**Francesc Calafell**

Spain

**Carla Calo**

Italy

**Lahcen Campbell**

Ireland

**Simona Candiani**

Italy

**Jun Cao**

China

**Cristian Capelli**

United Kingdom

**Tim Caro**

United States of America

**Martin Carr**

United Kingdom

**Bryan Carstens**

United States of America

**Jerome Casas**

France

**Antonio Castro**

Spain

**Isa Cavaco**

Sweden

**Stephen Cavers**

United Kingdom

**Stephane Chaillou**

France

**Yingguang Frank Chan**

Germany

**Belinda Chang**

Canada

**Ya-Ting Chao**

Taiwan

**Michel Chapuisat**

Switzerland

**Thierry Chardot**

France

**Gyaneshwer Chaubey**

Estonia

**Alan Cheng**

United States of America

**Changde Cheng**

United States of America

**Tzen-Yuh Chiang**

Taiwan

**Alberto Civetta**

Canada

**Marcus Clauss**

Switzerland

**Martin Coetzee**

South Africa

**David Collar**

United States of America

**Hélène Collin**

United Kingdom

**Gavin Conant**

United States of America

**Marty Condon**

United States of America

**Tim Connallon**

United States of America

**John Conran**

Australia

**Sofia Consuegra**

United Kingdom

**David Cooke**

United Kingdom

**Arielle Cooley**

United States of America

**Alan Cooper**

Australia

**Steven Cooper**

Australia

**Vaughn Cooper**

United States of America

**Miguel Corona**

United States of America

**Andrea Cosacov**

Argentina

**Sheena Cotter**

United Kingdom

**FPD Cotterill**

South Africa

**Marie-Agnes Coutellec**

France

**Robert Cowie**

United States of America

**Cymon John Cox**

Portugal

**Philip Cox**

United Kingdom

**Brian Crother**

United States of America

**Jose Cuesta**

Spain

**Christopher Cullis**

United States of America

**Cameron Currie**

United States of America

**Rute Da Fonseca**

Portugal

**Ann Daly United**

Kingdom

**Kavitha Damal**

United States of America

**Jakob Damgaard**

Denmark

**Igor Danilov**

Russian Federation

**Patrick Danley**

United States of America

**Liliana Davalos**

United States of America

**Charles Davis**

United States of America

**Jerrold Davis**

United States of America

**Jill De Jong**

United States of America

**A. P. Jason De Koning**

Canada

**Mario De La Fuente**

Chile

**Guillaume De Lafontaine**

Canada

**Thierry De Meeûs Burkina**

Faso

**Edivaldo De Oliveira**

Brazil

**Jacobus De Roode**

United States of America

**Janine Deakin**

Australia

**Rajib Deb**

India

**Melanie Debiais-Thibaud**

France

**Bernie Degnan**

Australia

**Susanne Den Boer**

Netherlands

**Fargette Denis**

France

**Annette Denzinger**

Germany

**Clio Der Sarkissian**

Denmark

**Philippe Deschamps**

France

**Maikon Di Domenico**

Brazil

**Anaid Diaz**

United Kingdom

**Muriel Dietrich**

Reunion

**Lubbert Dijkhuizen**

Netherlands

**David Dilcher**

United States of America

**Marc Dionne**

United Kingdom

**Martin Dohrmann**

Germany

**Steve Donnellan**

Australia

**Alex Dornburg**

United States of America

**Mario Dos Reis**

United Kingdom

**Julián Dowdall**

Germany

**David Drummond**

United States of America

**Louis du Plessis**

Switzerland

**Christine Dudgeon**

Australia

**Siobain Duffy**

United States of America

**Hannah Dugdale**

United Kingdom

**Robin Dunbar**

United Kingdom

**Walter Durka**

Germany

**Ingo Ebersberger**

Germany

**Vincent Eckhart**

United States of America

**Martin Edvardsson**

Australia

**Eran Elhaik**

United Kingdom

**Brian Ellis**

Canada

**John A Endler**

Australia

**Jan Engelstaedter**

Australia

**Lynn Epstein**

United States of America

**Peter Erdos**

Hungary

**Jacob Esselstyn**

United States of America

**William Etges**

United States of America

**Rampal Etienne**

Netherlands

**Isobel Eyres**

United Kingdom

**Hiroshi Ezura**

Japan

**Brant Faircloth**

United States of America

**Marie-Julie Favé**

Canada

**Jeffrey Fawcett**

Japan

**Jeffrey Feder**

United States of America

**Lars Fehre-Schmitz**

United States of America

**Chris Feldman**

United States of America

**Marie-Anne Felix**

France

**David Ferrier**

United Kingdom

**Ivan Fiala**

Czech Republic

**Konrad Fiedler**

Austria

**Renee Firman**

Australia

**John Fitzpatrick**

United Kingdom

**Thomas Flatt**

Switzerland

**Matthias Foellmer**

United States of America

**Luca Fontanesi**

Italy

**Kevin Foster**

United Kingdom

**Inês Fragata**

Portugal

**Lucia Franchini**

Argentina

**Steven Frank**

United States of America

**Bruno Frederich**

Belgium

**Jeffrey French**

United States of America

**Markus Friedrich**

United States of America

**Chris Friesen**

Australia

**Ville-Petri Friman**

United Kingdom

**Anthony Friscia**

United States of America

**Remy Froissart**

France

**Jérôme Fuchs**

France

**Andrzej Furman**

Turkey

**Monika Fuxreiter**

Hungary

**Kurt Galbreath**

United States of America

**Jose Galian**

Spain

**Juan Galindo**

Spain

**Milton Gallardo**

Chile

**Hugo Gante**

Switzerland

**Jessica Garb**

United States of America

**Fernando Garcia-Arenal**

Spain

**Andy Gardner**

United Kingdom

**Michael Garratt**

Australia

**Philippe Gaubert**

France

**Eric Gaucher**

United States of America

**Xuejun Ge**

China

**Kalle Gehring**

Canada

**Kerry Geiler-Samerotte**

United States of America

**Philippe Gerrienne**

Belgium

**Nelly Gidaszewski**

France

**Christopher Gilbert**

United States of America

**Joseph Gillespie**

United States of America

**Luis Gimenez**

United Kingdom

**Edmund Gittenberger**

Netherlands

**Jennifer Gleason**

United States of America

**Chaitanya S Gokhale**

New Zealand

**Elena Gomez**

United States of America

**Aida Gómez-Robles**

United States of America

**Xun Gong**

China

**Toni Gossmann**

United Kingdom

**Anjali Goswami**

United Kingdom

**Lena Göthlich**

Germany

**Yuval Gottlieb**

Israel

**Michael Gray**

Canada

**Eli Greenbaum**

United States of America

**Jennifer Greenwood**

Germany

**Emma Greig**

United States of America

**Sushma Grellscheid**

United Kingdom

**Laura Grieneisen**

United States of America

**Darren Griffin**

United Kingdom

**Linn Fenna Groeneveld**

Germany

**Mathieu Groussin**

France

**Christoph Grüter**

Switzerland

**Alena Gsell**

Germany

**Stephane Guindon**

New Zealand

**Joan Guinovart**

Spain

**Nicole Gunter**

Czech Republic

**Wen-Wu Guo**

China

**Vaclav Gvozdik**

Czech Republic

**Martin Haase**

Germany

**Rosemarie Haberle**

United States of America

**Markus Hafner**

United States of America

**Frank Hailer**

United States of America

**Ken Halanych**

United States of America

**Alex Hall**

Switzerland

**Heather Hallen-Adams**

United States of America

**Ben Halliwell**

Australia

**Kurt Hammerschmidt**

Germany

**Charles Nathan Hancock**

United States of America

**Thomas Hankeln**

Germany

**Joseph Hanly**

United Kingdom

**Andreas Hapke**

Germany

**Elisabeth Haring**

Austria

**Henry Harpending**

United States of America

**Eugene Harris**

United States of America

**Ellie Harrison**

United Kingdom

**Chie Hashimoto**

Japan

**Bernhard Hausdorf**

Germany

**Oliver Hawlitschek**

Germany

**Khaled Hazzouri**

United Arab Emirates

**Gerald Heckel**

Switzerland

**Andrew Heidel**

Germany

**George Heimpel**

United States of America

**Elizabeth Hellen**

United States of America

**Lars Hennig**

Switzerland

**Patrick Herendeen**

United States of America

**Russell Hermansen**

United States of America

**Greco Hernandez**

Mexico

**Matthew Herron**

Canada

**Robert Hershler**

United States of America

**Klemens Hertel**

United States of America

**Wolf Hirschmann**

Germany

**Amnon Hizi**

Israel

**Axel Hochkirch**

Germany

**Federico Guillermo Hoffmann**

United States of America

**Anneli Hoikkala**

Finland

**Barbara Holland**

New Zealand

**Phillip Hollingsworth**

United States of America

**Steven Hoofer**

United States of America

**Joaquin Hortal**

Spain

**Hsuan-Cheng**

Huang Taiwan

**Weini Huang**

Germany

**Dorothee Huchon**

Israel

**Alan Hudson**

Sweden

**Emilia Huerta-Sanchez**

United States of America

**Shih-Ying Hwang**

Taiwan

**Simone Immler**

Sweden

**Elina Immonen**

Sweden

**Yoshiharu Inoue**

Japan

**Manuel Irimia**

Canada

**Randall Irmis**

United States of America

**Md Sajedul Islam**

United States of America

**Takaya Iwasaki**

Japan

**Michelle Jacobs**

United States of America

**Rodolfo Jaffe**

Brazil

**David Jandzik**

Slovakia

**Robert Jansen**

United States of America

**Julie Jaquiery**

France

**Juan Pablo Jaramillo-Correa**

Mexico

**Armando Jardim**

Canada

**Martin Jastroch**

Germany

**Jeffrey Jensen**

Switzerland

**Enjalbert Jérôme**

France

**Huifeng Jiang**

China

**Liang Jiang**

China

**Pei-Luen Jiang**

Taiwan

**Zhao Jianmin**

China

**Yongfeng Jin**

China

**Louise Johnson**

United Kingdom

**Donna Jones**

United States of America

**Aslak Jørgensen**

Denmark

**Luca Jovine**

Sweden

**Daisuke Kageyama**

Japan

**Ingemar Kaj**

Sweden

**Yuichi Kameda**

Japan

**Aurelie Kapusta**

United States of America

**Takao Kasuga**

United States of America

**Masahiro Kato**

Japan

**Jim Kaufman**

United Kingdom

**Takeshi Kawakami**

Sweden

**Kazuhiko Kawasaki**

United States of America

**Alexander Keller**

Germany

**Barbara Keller**

Switzerland

**Irene Keller**

Switzerland

**Jennifer Kelley**

Australia

**Aurelie Khimoun**

France

**Yong-Kyu Kim**

United States of America

**Namshin Kim**

South Korea

**Benjamin King**

United States of America

**Kayla King**

United Kingdom

**David Kirk**

United States of America

**Andrew Kitchener**

United Kingdom

**Karl Kjer**

United States of America

**Stephan Koblmüller**

Austria

**Sarah Kocher**

United States of America

**Astrid Kodric-Brown**

United States of America

**Michael Hans Kohn**

United States of America

**Hanna Kokko**

Switzerland

**Dmitry Kopylov**

Russian Federation

**Vassiliki Koufopanou**

United Kingdom

**Elena Kramer**

United States of America

**Marcus Kronforst**

United States of America

**Matthias Kropf**

Austria

**Joachim Krug**

Germany

**Grit Kunert**

Germany

**Shigehiro Kuraku**

Japan

**Kaoru Kuriiwa**

Japan

**Verena Kutschera**

Germany

**Minna-Maarit Kytöviita**

Finland

**Karien Labuschagne**

South Africa

**Michael Landis**

United States of America

**Robert Lanfear**

Australia

**Enrique Lara**

Switzerland

**Dan Larhammar**

Sweden

**Peter Larsen**

United States of America

**Erica Lasek-Nesselquist**

United States of America

**Ane T. Laugen**

Finland

**Justin Lawrence**

United States of America

**Ester Lázaro**

Spain

**Arnaud Le Rouzic**

France

**Ellouise Leadbeater**

United Kingdom

**Larry Leamy**

United States of America

**Pei-Fen Lee**

Taiwan

**Michael Lee**

Australia

**Cheng-Ruei Lee**

Austria

**Luc Legal**

France

**Ben Lehner**

Spain

**Jussi Lehtonen**

Australia

**Tuomas Leinonen**

Finland

**Frederik Leliaert**

Belgium

**Tobias Lenz**

Germany

**Hannes Lerp**

Germany

**Fay-Wei Li**

United States of America

**Qing-Jun Li**

China

**Xiwen Li**

China

**Yang Li**

United States of America

**Jessica Liberles**

United States of America

**David Liberles**

United States of America

**Eva Liebau**

Germany

**Burton Lim**

Canada

**Doro Lindtke**

United States of America

**Charles Linkem**

United States of America

**Simone Linz**

Germany

**Thomas Lisney**

Canada

**Amy Litt**

United States of America

**Tom Little**

United Kingdom

**Cheng Liu**

China

**Peter Lockhart**

New Zealand

**Carla Jo Logan-Young**

United States of America

**Kevin Loope**

United States of America

**Xavier Lopez**

Spain

**Philippe Lopez**

France

**Jordi López-Pujol**

Spain

**Carlos Lopez-Vaamonde**

France

**Stefan Lötters**

Germany

**Stephen Lougheed**

Canada

**Jeffrey Lozier**

United States of America

**Andrea Luchetti**

Italy

**Sulan Luo**

China

**Stefan Lupold**

United States of America

**Andrew MacColl**

United Kingdom

**Gonzalo Machado-Schiaffino**

Germany

**D Luke Mahler**

United States of America

**Rafael Maia**

United States of America

**Irina Maljkovic Berry**

United States of America

**Richard Mally**

Norway

**Steven Manchester**

United States of America

**Mollie Manier**

United States of America

**Patrick Mardulyn**

Belgium

**Douglas Markle**

United States of America

**Peter Marko**

United States of America

**Therese Markow**

United States of America

**William Martin**

Germany

**Guillaume Martin**

France

**Julien Martinez**

United Kingdom

**Jesus Martinez-Padilla**

Spain

**John P Masly**

United States of America

**Nicholas Mason**

United States of America

**Daniel Maticzka**

Germany

**Yu Matsuura**

Japan

**Mike Maxwell**

United States of America

**Gerald Mayr**

Germany

**Karen D McCoy**

France

**Michael McGowen**

United Kingdom

**Joel McManus**

United States of America

**Mark McPeek**

United States of America

**Julie Meachen**

United States of America

**Richard H Meadow**

United States of America

**Raul Medina**

United States of America

**Jonathan Mee**

Canada

**Rita Mehta**

United States of America

**Carlo Meloro**

United Kingdom

**Vincent Merckx**

Netherlands

**Joachim Mergeay**

Belgium

**Sami Merilaita**

Finland

**Florian Mermillod-Blondin**

France

**Margarita Metallinou**

United States of America

**Neil Metcalfe**

United Kingdom

**Karen Meusemann**

Germany

**Axel Meyer**

Germany

**Justin Meyer**

United States of America

**Wolfgang Meyerhof**

Germany

**Theodore Meyers**

United States of America

**Leithen M'Gonigle**

United States of America

**Andy Michel**

United States of America

**Alexander Mikheyev**

Japan

**Alessandro Minelli**

Italy

**Rakesh Mishra**

India

**Michael Miyamoto**

United States of America

**Gaurav Moghe**

United States of America

**Vera Moiseenkova-Bell**

United States of America

**Babak Momeni**

United States of America

**Antonio Jose Monforte**

Spain

**Philippe Monget**

France

**Stefano Montanari**

Australia

**Francisco Montero**

Spain

**Michael Moody**

United States of America

**Paul Morris**

United States of America

**Edward Morrow**

United Kingdom

**Alan Moses**

Canada

**Adnan Moussalli**

Australia

**Ulrich Muller**

United States of America

**Tobias Müller**

Germany

**John Mulley**

United Kingdom

**Martha Munoz**

Australia

**Shona Murphy**

United Kingdom

**William Murphy**

United States of America

**Brent Murray**

Canada

**Navdeep Mutti**

United States of America

**Satoshi Namekawa**

United States of America

**Leanne Nash**

United States of America

**Nicolas Navarro**

France

**Andre Nel**

France

**Rafik Neme**

Germany

**Sterling Nesbitt**

United States of America

**Julienne Ng**

United States of America

**Chris Nice**

United States of America

**Caroline Nieberding**

Belgium

**Claus Nielsen**

Denmark

**Chen Niu**

United States of America

**Daniel Noble**

Australia

**Mohamed Noor**

United States of America

**Robert Nudds**

United Kingdom

**Juan Núñez-Farfán**

Mexico

**Suat Oezbek**

Germany

**Hiroyuki Ogata**

France

**Dario Ojeda Alayon**

Canada

**Brian Oliver**

United States of America

**Åke Olson**

Sweden

**Carrie Olson-Manning**

United States of America

**Denise O'Meara**

Ireland

**Juan Opazo**

Chile

**Claudia Patricia Ornelas-Garcia**

Mexico

**Pablo Orozco-Terwengel**

United Kingdom

**Guillermo Orti**

United States of America

**Lars Ostergaard**

United Kingdom

**Norman Owen-Smith**

South Africa

**Annalise Paaby**

United States of America

**Mark Pagel**

United Kingdom

**Maria Pala**

United Kingdom

**Ferran Palero**

France

**Andrei Papkou**

Germany

**Hanu Pappu**

United States of America

**Christine Parent**

United States of America

**Margot Paris**

France

**Daesik Park**

South Korea

**Kevin Parsons**

United Kingdom

**Mark Parthun**

United States of America

**Giuseppe Passarino**

Italy

**Bruce Patterson**

United States of America

**James L. Patton**

United States of America

**Joana Paupério**

Portugal

**James Pease**

United States of America

**Katia Pellegrino**

Brazil

**Carlos Pena**

Finland

**Osnat Penn**

United States of America

**Deepak Pental**

India

**Alan Pepper**

United States of America

**John Pepper**

United States of America

**Luisa Pereira**

Portugal

**Guy Perriere**

France

**George Perry**

United States of America

**Xavier Pico**

Spain

**Marcio Pie**

Brazil

**William Piel**

United States of America

**Davide Pisani**

United Kingdom

**Miroslav Plohl**

Croatia

**Grant Pogson**

United States of America

**Gianluca Polgar**

Brunei

**John Pool**

United States of America

**Anthony Poole**

New Zealand

**Emmanuelle Porcher**

France

**Daniele Porretta**

Italy

**Calvin Porter**

United States of America

**Megan Porter**

United States of America

**Daniel Portik**

United States of America

**Jaime Potti**

Spain

**Jan Yde Poulsen**

Norway

**Christine Pourcel**

France

**Thomas Powell**

United States of America

**Peter Prentis**

Australia

**Jill Preston**

United States of America

**Christopher Previti**

Germany

**Jonathan Price**

United States of America

**Tom Price**

United Kingdom

**Elizabeth Pringle**

United States of America

**Kathleen Prudic**

United States of America

**Jasna Puizina**

Croatia

**Benoit Pujol**

France

**Christian Rabeling**

United States of America

**Ute Radespiel**

Germany

**Jacek Radwan**

Poland

**Rahul Raghavan**

United States of America

**Steven Ramm**

Germany

**Pedro Range**

Portugal

**Vincent Ranwez**

France

**David Ray**

United States of America

**Suvendra Ray**

India

**Sushma Reddy**

United States of America

**Rosemary Redfield**

Canada

**Alexandre Reis Percequillo**

Brazil

**David L. Remington**

United States of America

**Jana Repkova**

Czech Republic

**Dirk Repsilber**

Sweden

**Paula Reyes-Herrera**

Italy

**Ângela Ribeiro**

Portugal

**Oldrich Rican**

Czech Republic

**Stefan Richter**

Germany

**Fabio Rindi**

Italy

**Christian Robert**

France

**Terence J Robinson**

South Africa

**Alfred Roca**

United States of America

**Luiz Rocha**

United States of America

**Nicolas Rodrigue**

Canada

**Igor Rogozin**

United States of America

**Ana Rojas**

Spain

**Antonis Rokas**

United States of America

**Jonathan Rolland**

France

**David Rollinson**

United Kingdom

**Sophie Rommel**

Germany

**Howard Rosenbaum**

United States of America

**Andrew Ross**

United Kingdom

**Omar Rota-Stabelli**

Italy

**Candy Rowe**

United Kingdom

**Kevin Rowe**

Australia

**Julio Rozas**

Spain

**Daniel Rozen**

Netherlands

**Nimrod Rubinstein**

United States of America

**Jasmin Ruch**

Germany

**Manuel Ruedi**

Switzerland

**Eduardo Ruiz-Sanchez**

Mexico

**Anna Runemark**

Sweden

**Anthony Russell**

Canada

**Danilo Russo**

Italy

**Assieh Saadatpour**

United States of America

**Carlos Saavedra**

Spain

**Keiko Sakakibara**

Japan

**Daniele Salvi**

Portugal

**Leah Samberg**

United States of America

**Alvaro San Millan**

United Kingdom

**Emilia Santos**

France

**Francisco Santos**

Portugal

**Mauro Santos**

Spain

**Alexandra Sá-Pinto**

Portugal

**Michael Savageau**

United States of America

**Hanno Schaefer**

Germany

**Helmut Schaschl**

Austria

**Christian Schlötterer**

Austria

**James Schnable**

United States of America

**Christian Schönbach**

Japan

**Frederick Schram**

United States of America

**Hannes Schuler**

United States of America

**Peter Schuster**

Austria

**Schraga Schwartz**

United States of America

**Erich Schwarz**

United States of America

**Roland Schwarz**

United Kingdom

**Matthew Scotch**

United States of America

**Catherine Searle**

United States of America

**Laure Segurel**

United States of America

**Ryan Seipke**

United Kingdom

**Jae Young Seong**

South Korea

**Irem Sepil**

United Kingdom

**Otto Seppälä**

Switzerland

**Robert Seyfarth**

United States of America

**Aaron Shafer**

Sweden

**Eugene Shakhnovich**

United States of America

**A. Jonathan Shaw**

United States of America

**Andrew Shedlock**

United States of America

**Lara Shepherd**

New Zealand

**Takahito Shikano**

Finland

**Mahmood Shivji**

United States of America

**John Shuey**

United States of America

**Dustin Siegel**

United States of America

**Julia Sigwart**

United Kingdom

**Ralph Simon**

Germany

**Luca Sineo**

Italy

**Tiratha Raj Singh**

India

**Jon Slate**

United Kingdom

**Adam Smith**

United States of America

**Gavin Smith**

Singapore

**James Sobel**

United States of America

**Jordi Solana**

Germany

**Emanuela Solano**

Italy

**Juan Jose Soler**

Spain

**Jingyuan Song**

China

**Ignacio María Soto**

Argentina

**Julien Soubrier**

Australia

**Dieter Spiteller**

Germany

**Peter F Stadler**

Germany

**Tanja Stadler**

Switzerland

**Klara Stefflova**

United States of America

**Sandra Steiger**

Germany

**Rike Stelkens**

United Kingdom

**Caitlin Stern**

United States of America

**John W. Stiller**

United States of America

**Tanja Strand**

Sweden

**Patrick Strutzenberger**

Germany

**Bing Su**

China

**Hans-Dieter Sues**

United States of America

**Shan Sun**

China

**Paul Sunnucks**

Australia

**Hitoshi Suzuki**

Japan

**Luc Swevers**

Greece

**Alfred Szmidt**

Japan

**Yasuoki Takami**

Japan

**Nobuto Takeuchi**

Japan

**Naoko Takezaki**

Japan

**Shohei Takuno**

Japan

**Gerard Talavera**

United States of America

**Kristiina Tambets**

Estonia

**Sithichoke Tangphatsornruang**

Thailand

**Pedro Tarroso**

Portugal

**Scott Taylor**

United States of America

**John Taylor**

Canada

**Ashley Teufel**

United States of America

**Anne Thielsch**

Germany

**Michael Thom**

United Kingdom

**Daniel Thomas**

Netherlands

**Paul Thomas**

United States of America

**Maria Tereza Thomé**

Brazil

**Dacheng Tian**

China

**Nicholas Tippery**

United States of America

**Takayuki Tohge**

Germany

**Koji Tojo**

Japan

**Haiyan Tong**

France

**João Filipe Tonini**

United States of America

**Arne Traulsen**

Germany

**Tom Tregenza**

United Kingdom

**Ludwig Triest**

Belgium

**Stephen Trumbo**

United States of America

**James Turner**

United Kingdom

**Xavier Turon**

Spain

**Alex Twyford**

United States of America

**Hildegard Uecker**

Austria

**Jeremy Ullmann**

Australia

**Nathan Upham**

United States of America

**Lawrence Uricchio**

United States of America

**Mario Vallejo-Marin**

United Kingdom

**Ines Van Bocxlaer**

Belgium

**Alexander van der Geer**

Netherlands

**Arie van der Meijden**

Portugal

**Joost van Heerwaarden**

Netherlands

**Simon van Noort**

South Africa

**Bettine van Vuuren**

South Africa

**John Vandenbergh**

United States of America

**Greg Vanlerberghe**

Canada

**Sandra Varga**

Finland

**Paris Veltsos**

United Kingdom

**Miguel Vences**

Germany

**Frederic Veyrunes**

France

**Linda Vigilant**

Germany

**Mark Viney**

United Kingdom

**Emma Vitikainen**

United Kingdom

**Thomas Von Rintelen**

Germany

**Christoph Vorburger**

Switzerland

**Niklas Wahlberg**

Finland

**Lutz Walter**

Germany

**Hongjian Wan**

China

**Qi Wang**

China

**Hurng-Yi Wang**

Taiwan

**Steve Wang**

United States of America

**Wen Wang**

China

**Erik Wapstra**

Australia

**Andrew Wargo**

United States of America

**Rachel Warnock**

United States of America

**Paul Wassarman**

United States of America

**Phillip Watts**

Finland

**Bonnie Webster**

United Kingdom

**Sonja Wedmann**

Germany

**Michèle Wegmann**

Switzerland

**Shu-Jun Wei**

China

**January Weiner**

Germany

**David Weisrock**

United States of America

**Steven Weiss**

Austria

**Mathias Wernet**

United States of America

**Sarah Werning**

United States of America

**Linda Wessel-Beaver**

Puerto Rico

**Jen White**

United States of America

**James Whitfield**

United States of America

**Martin Wiemers**

Germany

**John Wiens**

United States of America

**Stuart Wigby**

United Kingdom

**Shawn Wilder**

Australia

**Claus Wilke**

United States of America

**Eske Willerslev**

Denmark

**Christopher Willett**

United States of America

**Thomas Williams**

United States of America

**Tony Wilson**

United States of America

**Rachel Wiltshire**

United States of America

**Manuela Winkler**

Austria

**Michael Wiser**

United States of America

**Christian Woehle**

Germany

**Guinevere Wogan**

United States of America

**Diana Wolf**

United States of America

**Matthias Wolf**

Germany

**Yuri Wolf**

United States of America

**Rebecca Wood**

Australia

**April Wright**

United States of America

**Raquel Xavier**

Portugal

**Xuhua Xia**

Canada

**Fuliang Xie**

China

**Toshihiro Yamada**

Japan

**Marna Yandeau-Nelson**

United States of America

**Jin-Quan Yang**

China

**Jianrong Yang**

United States of America

**Keisuke Yoshida**

Japan

**Hon-Tsen Yu**

Taiwan

**Bisong Yue**

China

**Lorenzo Zane**

Italy

**Einat Zchori-Fein**

Israel

**Miriam Zelditch**

United States of America

**Aimin Zhang**

China

**De-Xing Zhang**

China

**Yuan-Ming Zhang**

China

**Yanjun Zhang**

China

**Chiyu Zhang**

China

**Mingli Zhang**

China

**Huabin Zhao**

China

**Li Zhao**

United States of America

**Yahui Zhao**

China

**Qing Zhou**

United States of America

**Renchao Zhou**

China

**Shunyi Zhu**

China

**Albert Zlotnik**

United States of America

